# Developmental Eye Movement test and dyslexic children: A pilot study with eye movement recordings

**DOI:** 10.1371/journal.pone.0200907

**Published:** 2018-09-07

**Authors:** Lionel Moiroud, Christophe Loic Gerard, Hugo Peyre, Maria Pia Bucci

**Affiliations:** 1 UMR 1141 Inserm - Université Paris Diderot, Robert Debré Hospital, Paris, France; 2 Child and Adolescent Psychiatry Department, Robert Debré Hospital, Paris, France; 3 Université Paris Diderot, Paris, France; Hangzhou Normal University, CHINA

## Abstract

The goal of this study is to explore eye movement recordings during the Developmental Eye Movement (DEM) test in dyslexic and non-dyslexic children. Thirteen children with dyslexia, 13 non-dyslexic chronological age- and IQ-matched children and 13 non-dyslexic reading age- and IQ-matched children were examined. Test C of the DEM test was performed with and without eye movement recordings (eye tracker by SuriCog). The results of the three groups were compared. Children with dyslexia and non-dyslexic children of equivalent reading age have significant longer fixation time and take longer to read Test C of the DEM test than non-dyslexic children of similar chronological age. A significant correlation was also found between the fixation time and the number of words read in one minute with the total time to read Test C of the DEM test. DEM test is a useful test for exploring the oculomotor behavior of dyslexic children during reading. The maturation of cortical structures controlling fixation capability appears to play a crucial role in reading skills.

## Introduction

Dyslexia has its origins in 1887 when Rudolf Berlin, an ophthalmologist, described it as a reading disorder [1]. More than a century later, and despite intensive research on the subject, we still do not know the etiology of such pathology. Pavlidis [2] was the first to show that Greek dyslexic children had abnormal eye movements and he suggested that such abnormalities could be responsible for reading difficulties (longer and more numerous fixations, shorter amplitudes of saccades, more backward saccades and unstable fixation). Since then, numerous studies in various languages have confirmed these results. Rayner [3] studied English dyslexic children and reported saccades of small amplitude and longer fixation of durations. Among Italian dyslexic children, [4] observed longer and more numerous fixations. In German children dyslexic [5] showed a relationship between the slower reading speed and a greater number of saccades. [6] highlighted similar oculomotor deficiencies in a Chinese dyslexic population. Our group [7–9] reported atypical eye movement patterns in French dyslexic children, suggesting a deficiency in the visual attentional processing as well as an immaturity of the interaction between the saccade and the vergence systems. Based on these studies we advanced the hypothesis that abnormal eye movements play a major role in reading difficulties.

Clinical tests to explore and assess eye movement’s performance in dyslexia have been developed and widely used by clinicians. One of these is the Developmental Eye Movement test (DEM) developed by Garzia et al. [10]. The test comprises two test cards (Texts A and B) with of 40 single numbers in each card that are arranged into two vertical columns of 20 numbers. Tests A and B are used to determine the automaticity of reading vertically-aligned numbers. In the third test card (Text C) the same 80 numbers are horizontal aligned (16 rows of 5 numbers each). According to the first DEM study [10] the Text C explores saccadic eye movement’s capability of the child. The DEM test is considered the best method to assess clinical indirect saccadic eye movements. It allows taking into account a potential difficulty at rapid naming (*i*.*e*. the ability to verbalize what we see) that is believed to have a predictive value of the child’s reading performance [11]. This test also allows discriminating oculomotor disorders from phonological and/or lexical disorders [12,13]. The DEM test is a simple way to explore the saccades associated with reading in school age children. However, Ayton et al. [14] have questioned its validity. These authors did not find any correlation between the DEM test performance and saccadic parameters (accuracy, latency, speed) although they established a strong correlation with the reading performance. It should also be noted that other publications [11,15] show that the results at the DEM test do not correlate with poor saccadic performance or other oculomotor symptoms. However, for all these authors, the DEM test is useful for judging the reading performance and speed of visual processing. To our knowledge, no study focuses on a population of dyslexic children, while the DEM test was originally designed for children with reading disorders. We also noted that no study has recorded eye movements with a video-oculography while performing the DEM test.

We made the hypothesis that an objective analysis of eye movements while the subjects are performing the test DEM (Text C) would highlight visual deficits in dyslexic children. As the majority of clinical tests used by orthoptists the DEM test evaluates oculomotor performances without an objective eye movements recording; for this reason we wonder to objectively evaluate the reliability of the DEM test by employing an eye tracker. For instance, duration fixation could be an important parameter which will discriminate dyslexic versus non-dyslexic children. We examined a group of children with dyslexia and compared these data with those of a group of non-dyslexic, reading age matched children and another group of non-dyslexic, chronological age matched children. Recall that oculomotor performances are age-dependent [16] and given that in dyslexic children has been reported an immaturity of the cortical structures triggering eye movements [7,9] in studies leading with reading and oculomotor capability on dyslexia it is essential to compare dyslexic children with both non-dyslexic reading age- and chronological age-matched children group. According to studies from other [17,18] and our group [7,9] this allow to explore the potential causality between some cognitive factors and reading difficulty.

## Methods

Thirteen dyslexic children from 7.5 to 12 years old (mean age: 10.4 ± 0.43 years) participated in the study. Dyslexic children were recruited from Robert Debré pediatric hospital, to which they had been referred for a complete evaluation of their dyslexia including an extensive examination of their neurological/psychological and phonological capabilities. For each child, we measured the time they required to read a text passage, assessed their general text comprehension, and evaluated their ability to read words and pseudo-words using the L2MA battery [19]. This is the standard test in France. It was developed by the “Centre de Psychologie appliquée de Paris” and is used to detect dyslexic populations. Inclusion criteria were scored on the L2MA which were more than two standard deviations from the mean, and a normal mean intelligence quotient (IQ, evaluated using the WISC-IV). Any hyperactivity deficit was excluded using the ADHD Rating Scale-parental report (ADHD-RS [20]). In more details, inclusion criteria were: (a) Age between 7–12 years; (b) Scores of L2MA battery beyond two standard deviations; (c) Score of Intelligence Quotient (IQ) evaluated with WISC-IV ranging between 80 and 115; (d) Normal visual acuity for distance vision and near vision (both eyes ≥10/10); (e) Absence of strabismus, amblyopia or degenerative pathology affecting eyes (cataract, scotoma, retinopathy); (f) Absence of any signs of hyperactivity or lack of coordination related to development; (g) Not taking drugs that could modify their visual behavior or perception of the subject; (e) Not suffering from neurological or mental disorders or disabilities that prevents a proper understanding of the tests.

Thirteen chronological and reading age-matched non-dyslexic children respectively of 10.3 ± 0.46 years old and 7 ± 0.24 years were also examined. The inclusion criteria were as follows: no known neurological or psychiatric abnormalities, no history of reading difficulty, no visual impairment, or difficulty with near vision. Also, IQ in controls was estimated on two subtests, one assessing their logic capability (matrix reasoning test), and one assessing their verbal capability (similarities test). Normal range for both tests is 10 ± 3 (Wechsler intelligence scale for children—fourth edition, 2004). The ELFE test (cogni-sciences, Grenoble) was used to measure the reading age of all children.

The investigation adhered to the principles of the Declaration of Helsinki and was approved by our Institutional Human Experimentation Committee (INSERM CEEI-IRB, n° 16–290). Written informed consent was obtained from the children’s parents after they were given an explanation about the experimental procedure.

### Visual evaluation

All children underwent a complete visual examination (showed in Table 1).

**Table 1:** Mean and standard error of convergence and divergence values at near distance.

	Convergence far (δ)	Divergence far (δ)	Convergence near (δ)	Divergence near (δ)	NPC (cm)
**Dyslexic children**	17 ± 1	4.8 ± 0.5	29 ± 3	13 ± 0.8	6.5±0.7
**Reading age matched children**	17 ± 1	6 ± 1	38 ± 1	12 ± 1.3	5±0
**Chronological age matched children**	17 ± 1	3.4 ± 0.2	32 ± 0.25	13 ± 0.7	6±0.5

For all children the corrected visual acuity was normal (≥ 20/20). All children had normal binocular vision (measured with the TNO test). The near point of convergence (NPC) was normal for all children. Amplitudes of vergences (convergence and divergence) were measured at far and near distance using a base-in and a base-out prism bar and were in the normal range.

The DEM test (texts A, B and C) was also performed by each child (without eye movement recording).

### DEM test and eye movement recording

**Fig 1:**
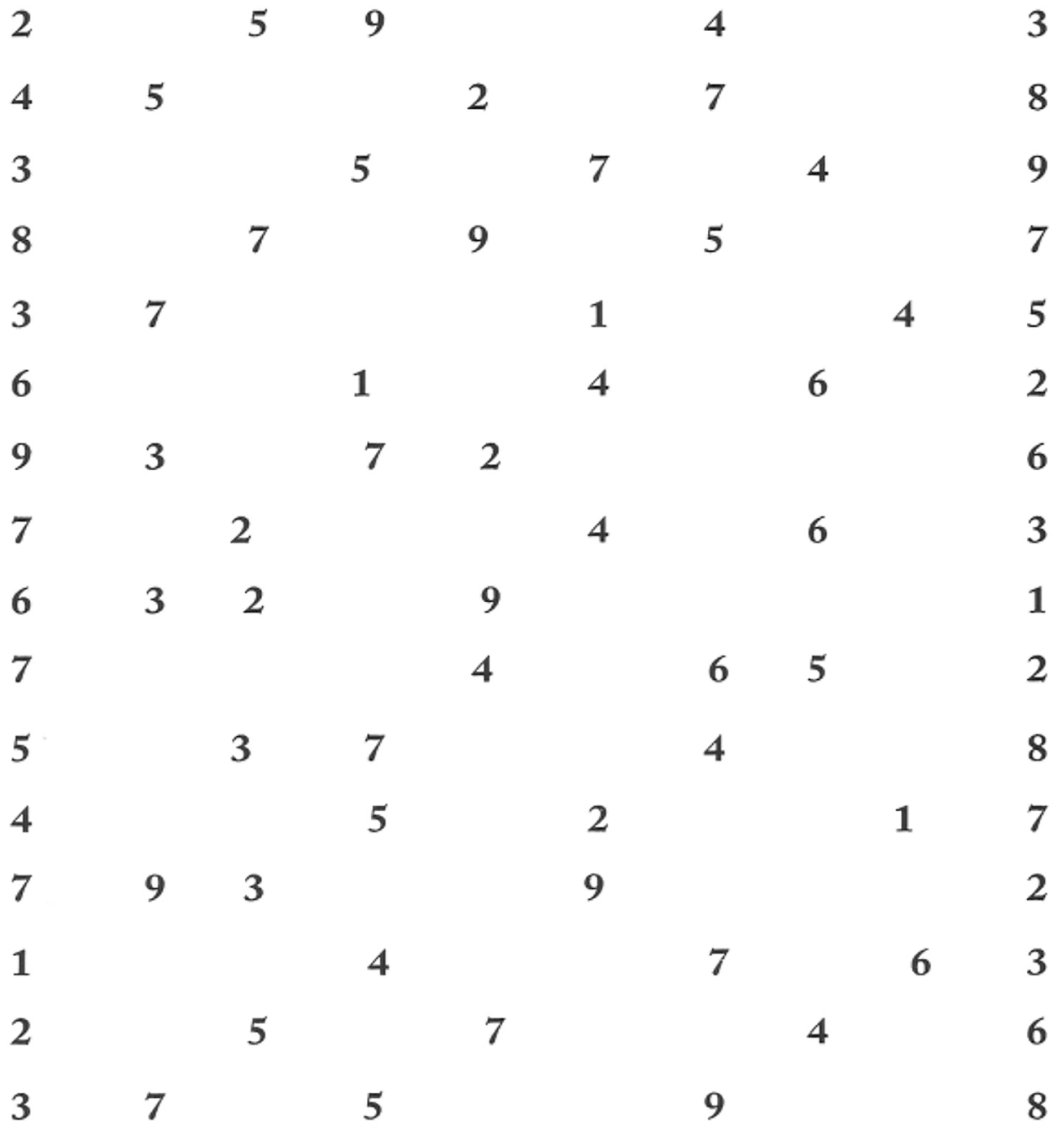
Test C of the DEM test.

Text C of the DEM test was presented on a 22″ PC screen with a resolution of 1920×1080 and a refresh rate of 60 Hz. The child was seated in a chair in a dark room, with his/her head stabilized by a forehead and chin support. Viewing was binocular; the viewing distance was 40 cm. Calibration was done at the beginning of reading Text C of the DEM test. During the calibration procedure, the children were asked to fixate a grid of 13 points (diameter 0.5 deg) mapping the screen (for details [21]). Afterwards, the child was invited to read aloud the numbers of Test C of the DEM test (see Fig 1) as quickly as possible, keeping his/her head stationary and continuing to the end regardless of mistakes along the way.

Eye movements were recorded binocularly; horizontal and vertical eye positions were recorded independently and simultaneously for each eye with the EyeBrain T2 (SuriCog), an eye-tracking device CE-approved for medical applications. Recording frequency for both eyes was set up to 300 Hz.

Test C of the DEM test with and without eye movement recordings was performed randomly by each child. Children were asked to read more precise and quickly as possible the numbers from left to right side line by line horizontally.

### Data analysis

Calibration factors for each eye were determined from the eye positions during the calibration procedure (see Bucci et al. [7] for details). The number and amplitude of saccades (prosaccades, from left to right) and of regressive saccades (backward saccades, from right to left) and the duration and number of fixations between each saccade were analyzed. For each child, we measured the total time to read Text C of the DEM test in both recordings, with and without eye movements.

### Statistical analysis

A linear regression model was used in which the dependent variable was the number of words read during 1 minute at the ELFE test and the fixation of durations between the saccades, and the predictor variable was the time used to read Text C of the DEM test in the eye movement recording session for all children tested. An analysis of variance (ANOVA) was also performed with groups as inter-subject factor and the oculomotor parameters as within-subject factors. Bonferroni post-hoc comparisons were employed. The effect of a factor is significant when the *p*-value is below 0.05.

## Results

**Fig 2:**
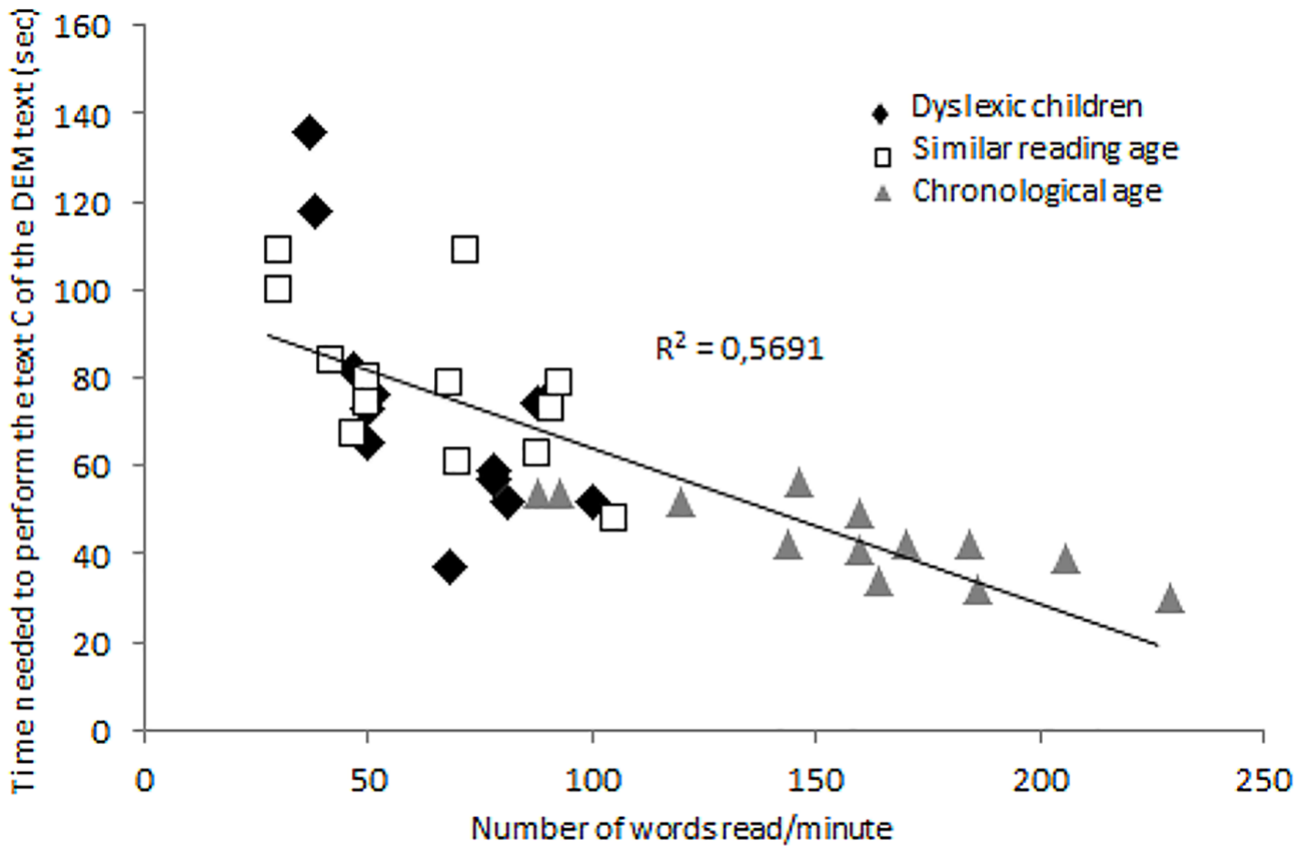
Correlation between the time needed to read Text C of the DEM test (in seconds) and the number of words read during 1 minute at the ELFE test for each child.

Fig 2 shows the correlation between the number of words read per minute at the ELFE test and the time (in seconds) for reading Text C of the DEM test for all children tested. For each group of children there was a significant negative correlation (r = -0.65, *p* < 0.01; r = -0.59, *p* < 0.03; r = -0.81, *p* < 0.001, respectively for dyslexic, reading age- and chronological age-matched children group): children reading slower need more time to read Text C of the DEM test.

**Fig 3:**
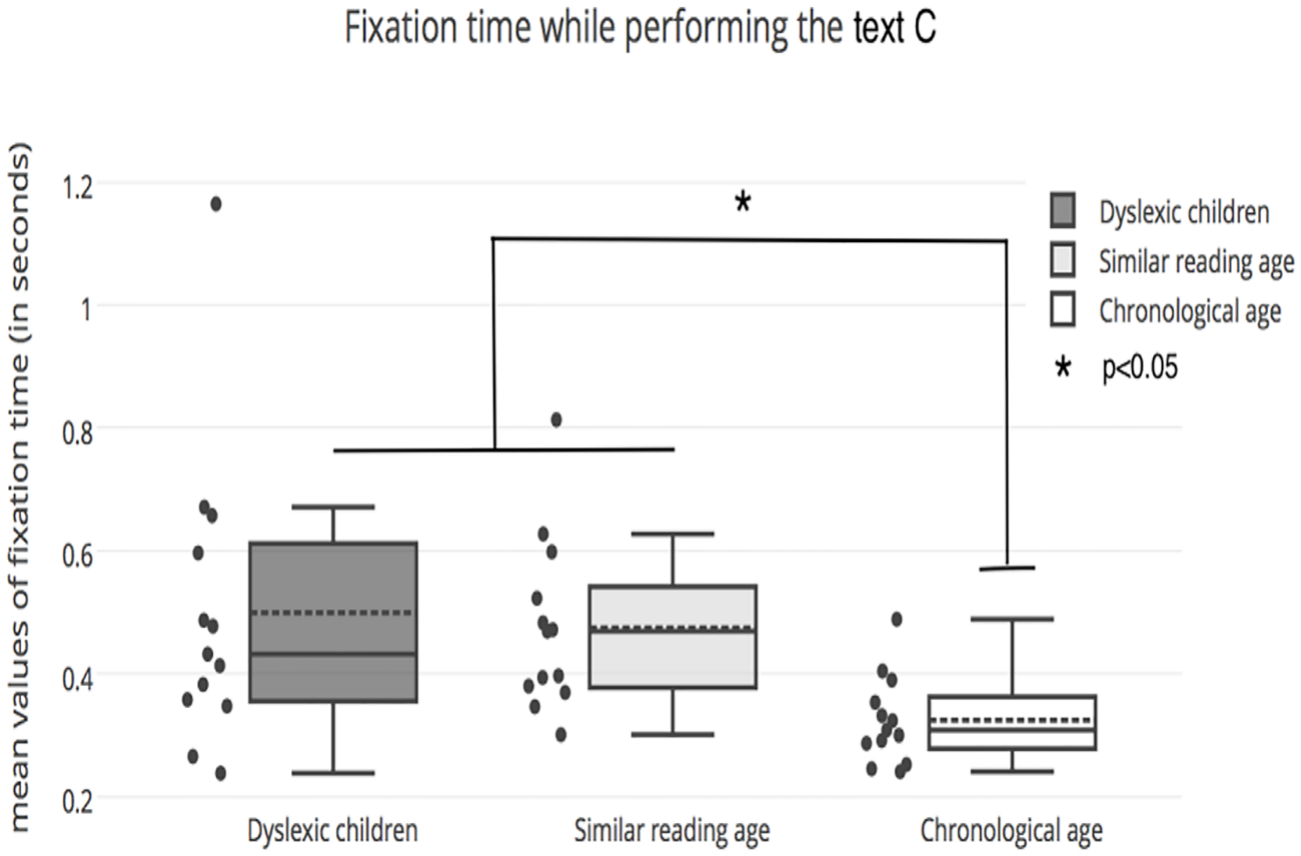
Mean values of fixation time (in seconds) for each group of children tested.

ANOVA also reported (Fig 3) a significant difference in the three groups of children for the fixation time while reading Text C of the DEM test (F_(2,36)_ = 4.23, *p* <0.05); indeed, Bonferroni correction reported that dyslexic children and children of similar reading age had a longer fixation time than the group of children of similar chronological age (p <0.01 and *p* <0.02, respectively).

**Fig 4:**
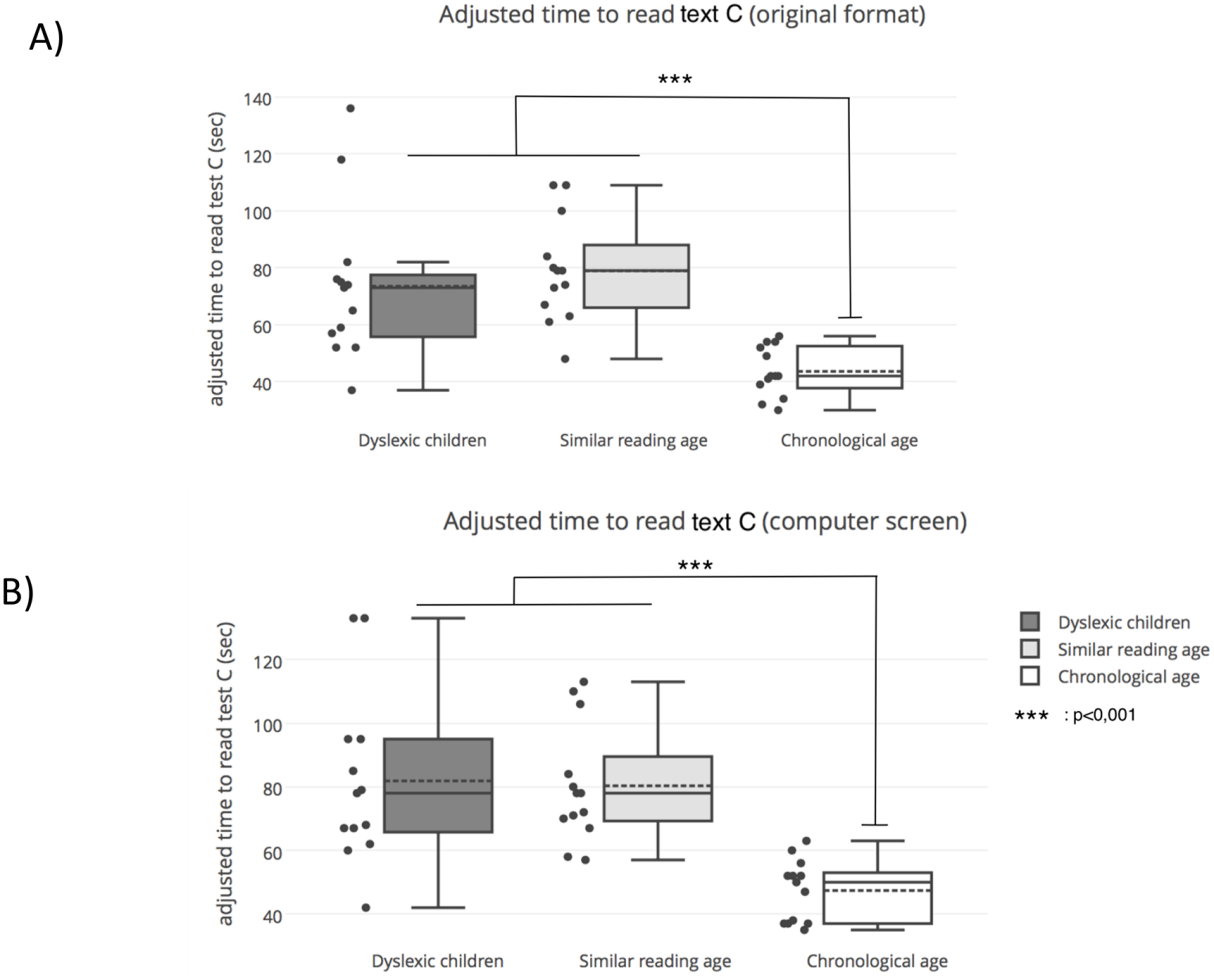
Adjusted time (in seconds) used to perform Text C of the DEM test without (A) and with (B) eye movement recordings for each group of children tested.

The total time to read Text C of the DEM test without and with eye movements recording is shown in Fig 4A and 4B, respectively. The ANOVA showed a significant difference in each of these sessions (F_(2,36)_ = 12.35, *p* <0.0005 and F_(2,36)_ = 12.73, *p* <0.0005, respectively). Bonferroni correction reported that dyslexic children and the reading age-matched children took longer time to perform this task with respect to the chronological age-matched children group (all *p* < 0.0001).

**Fig 5:**
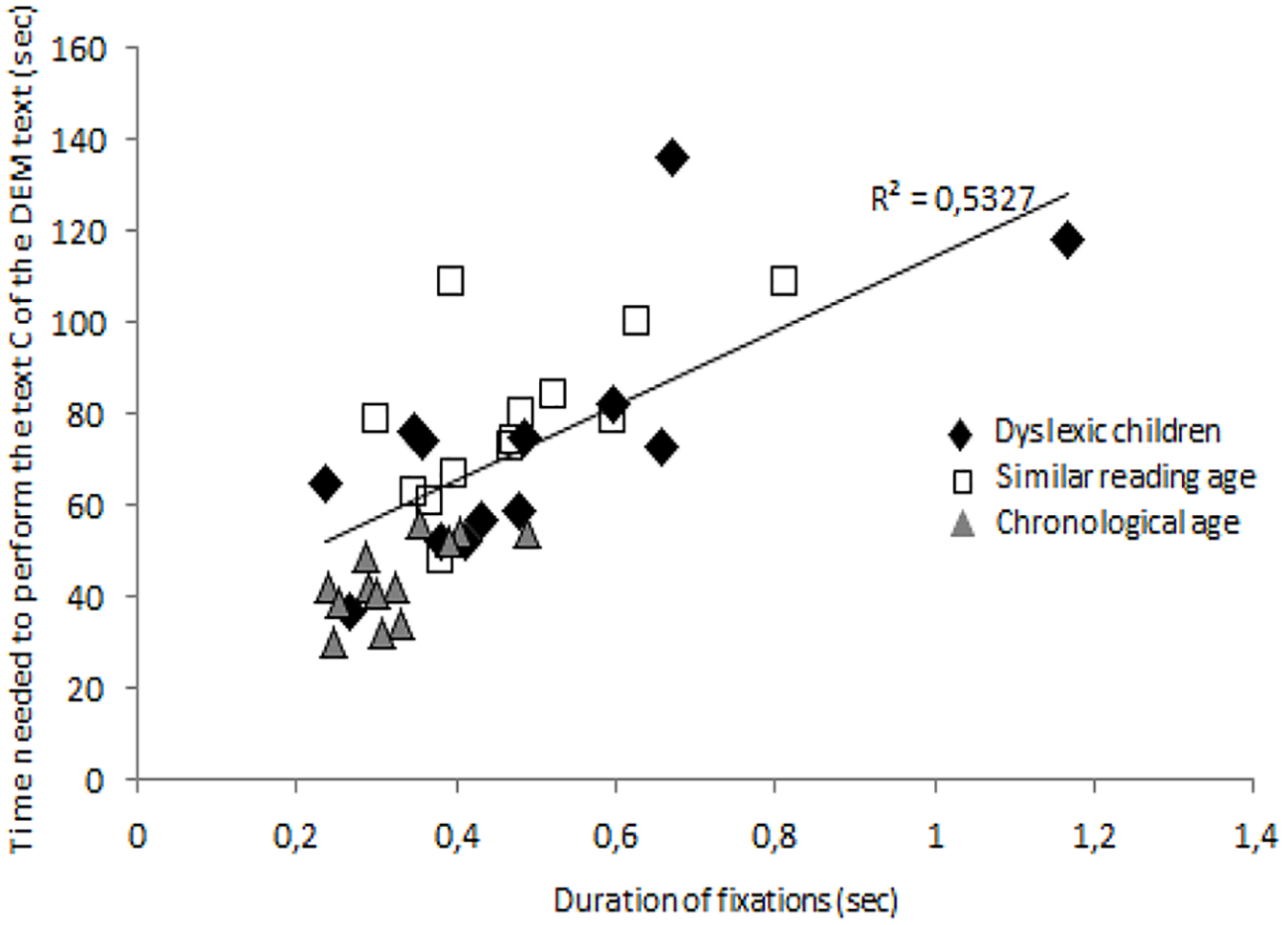
Correlation between the mean duration of fixation (in seconds) and total time to read Text C of the DEM test (in seconds) for each child tested.

Finally we also found a positive correlation (Fig 5) between the duration of fixation and the total time needed to read Text C of the DEM test for all children tested. For each group of children there was a significant positive correlation (r = 0.73, *p* < 0.005; r = 0.62, *p* < 0.02; r = 0.67, *p* < 0.01, respectively for dyslexic, reading age- and chronological age-matched children group).

ANOVA failed to show any significant difference between the three groups of children for the number of fixations (F_(2,36)_ = 2.45, *p* = 0.09), for the number and the amplitude of prosaccades (F_(2,36)_ = 0.88, *p* = 0.42 and F_(2,36)_ = 2.01, *p* = 0.85, respectively) and for the number and the amplitude of backward saccades (F_(2,36)_ = 1.64, *p* = 0.21 and F_(2,36)_ = 1.05, *p* = 0.34, respectively), as shown in Table 2.

**Table 2:** Mean and standard errors of the number of fixations, numbers and amplitude of prosaccades and of backward saccades for each group of children tested.

	Fixations Numbers	Prosaccades		Backward saccades	
		Numbers	Amplitude (deg)	Numbers	Amplitude (deg)
**Dyslexic children**	150 ± 14	127 ± 17	3 ± 0.1	23 ± 5	2.5 ± 1.2
**Reading age-matched children**	134 ± 10	120 ± 15	3 ± 0.1	14 ± 2	2.2 ± 1.1
**Chronological age-matched children**	114 ± 7	127 ± 9	3 ± 0.1	12 ± 2	2.0 ± 1.5

## Discussion

We reported oculomotor similarities between the group of children with dyslexia and the group of non-dyslexic, reading age-matched children. The main findings of this study are as follows: (i) Dyslexic children and non-dyslexic, reading age-matched children have a similar fixation of durations that is significantly longer than that of non-dyslexic chronological age-matched children. (ii) Dyslexic children and non-dyslexic reading age-matched children take a longer time to read Text C of the DEM test than non-dyslexic, chronological age-matched children. (iii) There is also a significant negative correlation between the number of words read per minute at the ELFE test and the duration fixation with the total time needed to read Text C of the DEM test. These findings will be discussed individually below.

### Longer fixation of durations in dyslexic children and non-dyslexic reading age-matched children

In the present study we have shown that the fixation of durations was significantly longer in dyslexic children than in non-dyslexic, chronological age-matched children, while no difference was found for the number and the amplitude of saccades. It is well known that dyslexic children showed smaller and frequent saccades during reading a text only [4,22] and not while performing saccades to visual targets [23,24]. In the present study during the DEM test children had not to read a text, consequently it was quite normal that no difference was found in saccade performance but only in fixation ability.

Recall that several studies conducted on children during reading a text in different languages (French [22], English [3], Italian [4], Greek [25], Chinese [6] and German [5] reported longer fixation of durations. However, we have to point out that in the present study children did not read words but numbers; consequently the length of words, and the difficulty of the text did not interfere with such longer fixation of durations. The abnormal duration of fixation is independent from the reading task. Previous work [9,26] reported a poor quality of fixation in dyslexic children independently from any reading activity, most likely due to attentional deficits. Even if dyslexic children did not have attentional deficiencies (see Methods section) we suggest that poor attentional abilities could be at the origin of such poor fixation control.

In agreement with previous studies [11,14,15] we have also shown that the time taken by the children (dyslexic and non dyslexic) to read Text C of the DEM test was significantly correlated with the speed of reading a text (ELFE). Taken together, these results suggest that there is a link between visual processing, performance of verbalization and the DEM test to clinically assess the reading performance of children with and without reading deficiencies. This hypothesis contrasts with the theory / hypothesis of Medland et al. [27], for whom the abnormal performance of saccades observed in dyslexic children would be the effect rather than the cause of reading difficulties; consequently, for these authors the DEM test is not useful to detect eye movement difficulties in dyslexia.

### DEM test and eye movements

The DEM test is regularly used by clinicians to screen children with reading difficulties. Northway [28] suggested that the DEM test could be useful to explore the benefit of colored overlays in dyslexic children because it allows determining the presence or not of eye movement deficit, while Powers [29] suggested that the DEM test could just give information on eye behavior during reading, and Ayton et al. [14] showed that there was no correlation between DEM scores and quantitative eye movement measures.

We have to point out that none of these studies recorded objectively eye movements while performing the DEM test, as we did in the present study. Our results showed that there was no significant difference between the three groups of children in terms of saccade amplitude and on the number of backward saccades while reading Text C of the DEM test; indeed we found a difference in the duration of fixations only. Note that the reading process is more complex than simple saccadic movements between two points; and as Rayner [3] suggested, it is during the fixation that a child understands the meaning of the word he/she is reading. Consequently, researchers need to focus on the fixation performance in dyslexic as well as in non-dyslexic children, and not on saccadic performance only.

### Similar oculomotor behavior in dyslexic and non-dyslexic reading age-matched children

The DEM performance is age-dependent; consequently it is normal to find similar results in dyslexic children and in non-dyslexic reading-age matched children. Both groups of children had the same features when reading numbers and when they read letters: several longer durations of fixations in relation with their reading capabilities. Our group [7,9] advanced the hypothesis that an immaturity of the oculomotor system in dyslexic children could be the cause of such poor fixation capabilities, in line with the findings of Luna et al. [16] showing that the quality of visual fixation in younger children is poor and improves until adolescence. The present study shows longer fixation durations at the DEM test that is similar in dyslexic children and in reading age matched non-dyslexic children. Further studies will need to explore further whether longer fixation durations are the cause or the effect of reading impairment in dyslexia.

How can we improve the quality of fixations in dyslexic children? Bosse et al. [30] suggested that dyslexics have a reduced visuo-attentional window resulting in a limitation of the number of letters that can be processed in parallel, leading to a greater number of fixations and shorter saccades during reading. The improvement of the visuo-attentional window could be useful to dyslexics for improving the quality of fixations. Studies examining training of visual attentional capabilities in dyslexic children will be necessary to explore further such issue.

## Conclusion

Even if the number of children tested in the present study is small, we reported that the use of an eye tracker allows one to show that the DEM test is a useful tool to show poor fixation capabilities in dyslexic children.
